# Extravascular implantable cardioverter-defibrillator implantation strategies following transvenous or subcutaneous device infections: a case series

**DOI:** 10.1093/ehjcr/ytag507

**Published:** 2026-07-14

**Authors:** Satoshi Ono, Satoshi Oka, Mitsuru Wada, Kohei Ishibashi, Akinori Wakamiya, Nobuhiko Ueda, Kosuke Nakasuka, Kengo Kusano

**Affiliations:** Department of Cardiovascular Medicine, National Cerebral and Cardiovascular Center, 6-1 Kishibeshimmachi, Suita, Osaka 564-8565, Japan; Department of Cardiovascular Medicine, National Cerebral and Cardiovascular Center, 6-1 Kishibeshimmachi, Suita, Osaka 564-8565, Japan; Department of Cardiovascular Medicine, National Cerebral and Cardiovascular Center, 6-1 Kishibeshimmachi, Suita, Osaka 564-8565, Japan; Department of Cardiovascular Medicine, National Cerebral and Cardiovascular Center, 6-1 Kishibeshimmachi, Suita, Osaka 564-8565, Japan; Department of Cardiovascular Medicine, National Cerebral and Cardiovascular Center, 6-1 Kishibeshimmachi, Suita, Osaka 564-8565, Japan; Department of Cardiovascular Medicine, National Cerebral and Cardiovascular Center, 6-1 Kishibeshimmachi, Suita, Osaka 564-8565, Japan; Department of Cardiovascular Medicine, National Cerebral and Cardiovascular Center, 6-1 Kishibeshimmachi, Suita, Osaka 564-8565, Japan; Department of Cardiovascular Medicine, National Cerebral and Cardiovascular Center, 6-1 Kishibeshimmachi, Suita, Osaka 564-8565, Japan

**Keywords:** Cardiovascular implantable electronic device-related infection, Extravascular implantable cardioverter-defibrillator, Subcutaneous implantable cardioverter-defibrillator, Transvenous implantable cardioverter-defibrillator

Learning points

- Implantation of an extravascular implantable cardioverter-defibrillator (EV-ICD) following infection of a transvenous or subcutaneous ICD is feasible.
- Intermuscular EV-ICD implantation may be a beneficial option in patients with a small body size and extremely thin skin.
- When converting from a subcutaneous ICD to an EV-ICD following infection, midline incisions can be placed without direct overlap; however, partial overlap can occur in the lateral thoracic region.

## Introduction

Cardiovascular implantable electronic device-related infection is potentially life-threatening, particularly in cases of infective endocarditis. Endocarditis and bacteraemia occur in 22%–54% of transvenous implantable cardioverter-defibrillator (TV-ICD) infections,^1,2^ whereas they are rarely associated with subcutaneous ICD (S-ICD) infections,^3,4^ underscoring a clear distinction between intravascular and extravascular systems.^5^ Previous reports have demonstrated that S-ICD implantation is feasible even for patients with a small body size.^6^ However, the large generator size and suprasternal lead implantation may increase the risk of skin erosion, particularly in patients with thin subcutaneous tissue.

A novel extravascular ICD (EV-ICD) system, with a smaller generator size than the S-ICD system and substernal lead implantation technology,^7^ may be a useful alternative following extraction of a TV- or S-ICD system owing to infections, offering a potential advantage of an extravascular system with a reduction in skin tension and stress. However, reports on EV-ICD implantation after infection of a TV-ICD or S-ICD remain limited.^8–10^

Herein, we present a case series of EV-ICD implantation following TV- or S-ICD infections, considering optimal strategies to prevent reinfection.

## Summary figure

**Figure:**
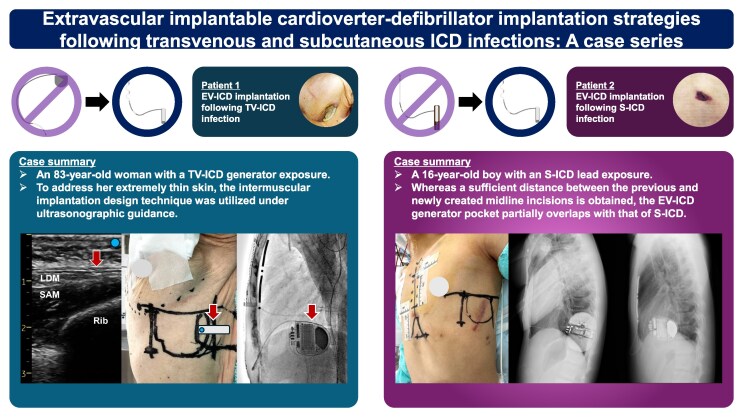


## Case series

### Patient 1

**Figure 1:**
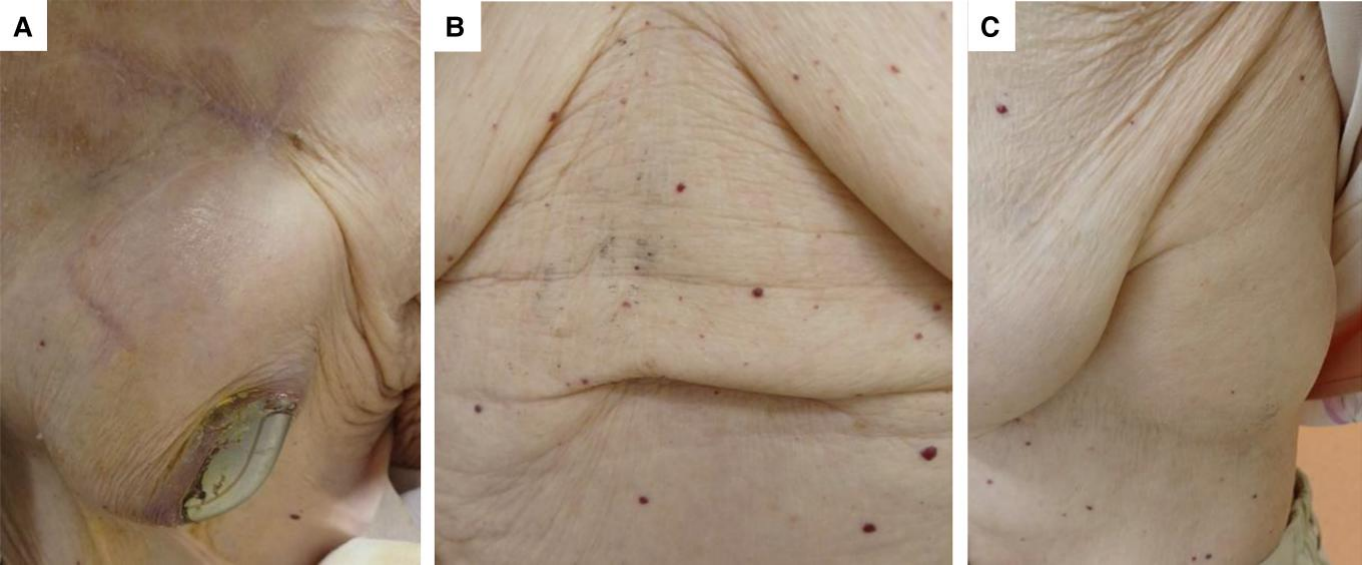
Pre- and post-operative body surface findings in a case of an EV-ICD implantation following TV-ICD infection. Pictures of pre-operative body surface findings demonstrating a TV-ICD generator exposure and device-pocket infection owing to extremely thin subcutaneous tissue (*A*), post-operative appearance of the midline incision (*B*), and the EV-ICD generator implantation site (*C*) 1 month after the implantation. EV-ICD, extravascular implantable cardioverter-defibrillator; TV-ICD, transvenous implantable cardioverter-defibrillator.

An 83-year-old woman with hypertrophic cardiomyopathy underwent TV-ICD implantation for primary prevention 18 years ago owing to a history of syncope, a family history of sudden cardiac death, and non-sustained ventricular tachycardia. She was hospitalized for generator exposure and a device-pocket infection (*Figure 1*). The TV-ICD system was successfully extracted using a locking stylet, an 11 Fr controlled-rotation dilator sheath set, and a 12 Fr excimer laser sheath. She requested ICD reimplantation because of her history of anti-tachycardia pacing (ATP) therapy for sustained ventricular tachycardia; however, she was considered at high risk of recurrent device exposure and infection given her small body size (height, 143 cm; body weight, 34 kg; body mass index, 16.3 kg/m^2^) and extremely thin subcutaneous tissue. Therefore, an EV-ICD system was selected as the reimplantation device, given its smaller generator size compared with the S-ICD system and absence of intravascular components.

**Figure 2:**
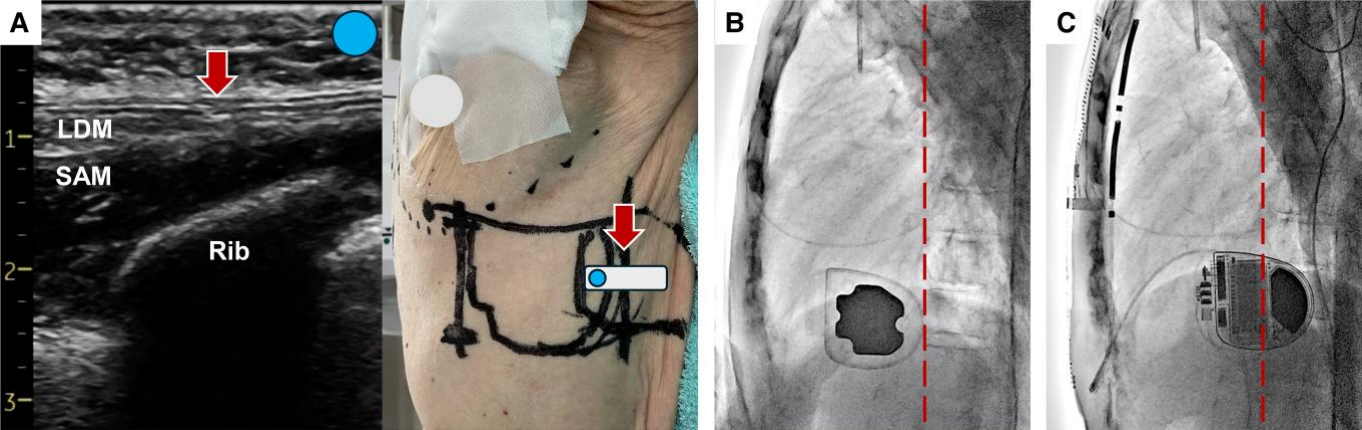
Preprocedural extravascular implantable cardioverter-defibrillator (EV-ICD) implantation design utilizing ultrasonographic assessment. Ultrasonography-guided preprocedural implantation design was performed. After identifying the anterior border of the latissimus dorsi muscle (LDM) (red arrow), intermuscular EV-ICD implantation between the LDM and the serratus anterior muscle (SAM) was planned (*A*). Compared to the recommended generator position (*B*) determined by the posterior border of the left ventricular silhouette (red-dotted line) on the left lateral fluoroscopic view, the EV-ICD generator was implanted posteriorly (*C*).

Following 2 weeks of antibiotic treatment, EV-ICD implantation was performed under general anaesthesia. To address the high risk of device exposure, the implantation design was planned by applying an intermuscular pocket creation technique originally described for S-ICD implantation.^6,11^ Ultrasonography was used to identify the anterior border of the latissimus dorsi muscle, which facilitated the planning of the generator position and incision line (*Figure 2A*). The substernal lead was implanted through the standard left-sided substernal space, yielding an acceptable R-wave amplitude of 2.4 mV on the ring 1–ring 2 vector without P-wave oversensing. Based on the pre-operative design, the EV-ICD generator was successfully implanted between the serratus anterior and latissimus dorsi muscles. Despite posterior implantation, as compared with the recommended position determined by the posterior wall silhouettes of the left ventricle in the left lateral view (*Figure 2B* and *C*),^12^ the induced ventricular fibrillation was successfully defibrillated with a 15 J shock, confirming an adequate safety margin. During the 11-month follow-up period, no reinfection, device exposure (*Figure 1B* and *C*), and ATP or shock therapies were observed. On the ring 1–ring 2 vector, the lead parameters remained within the clinically acceptable ranges, with an R-wave amplitude of 3.7 mV and an impedance of 475 Ω.

### Patient 2

**Figure 3:**
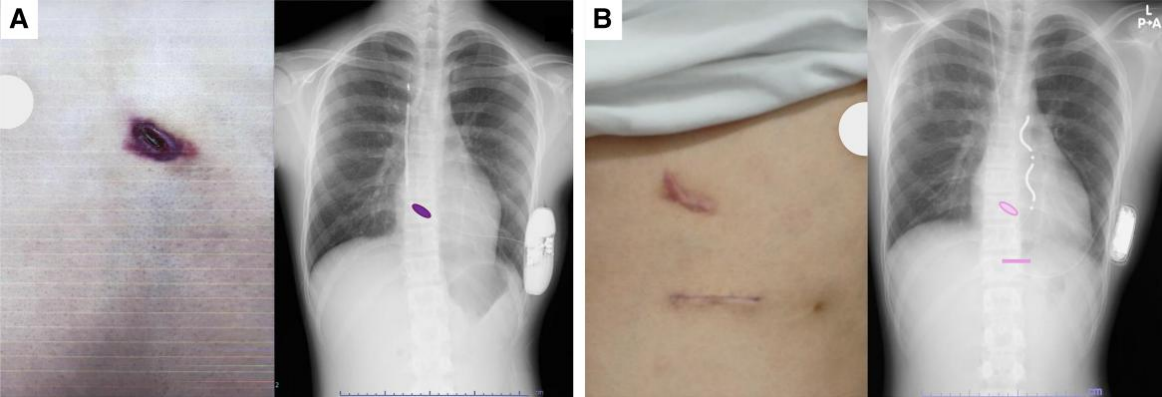
Pre- and post-procedural parasternal body surface appearances and chest radiographs in the case of EV-ICD implantation following S-ICD infection. An exposed subcutaneous lead at the midline infected incision site and a posteroanterior chest radiograph prior to the S-ICD extraction are shown (*A*). A post-operative picture of the parasternal body surface appearance and a radiograph after 1 month showed successful EV-ICD implantation with a sufficient distance between the previous and the newly created incisions (*B*). EV-ICD, extravascular implantable cardioverter-defibrillator; S-ICD, subcutaneous implantable cardioverter-defibrillator.

A 16-year-old boy was transferred to our hospital for EV-ICD implantation after S-ICD extraction and 2 weeks of antibiotic treatment for an infection around the midline incision site with lead exposure (*Figure 3A*). The patient experienced an out-of-hospital ventricular fibrillation-related cardiopulmonary arrest and was successfully resuscitated using an automated external defibrillator. A subsequent diagnosis of hypertrophic cardiomyopathy led to S-ICD implantation for secondary prevention 8 months prior to the infection. A standard left-parasternal subcutaneous lead position could not be used because of T-wave oversensing; therefore, a right parasternal implantation partially across the sternum successfully passed the criteria for S-ICD sensing performance. As a repeat episode of ventricular fibrillation treated with appropriate shock therapy was observed 5 months before device extraction, ICD reimplantation was absolutely recommended.

**Figure 4:**
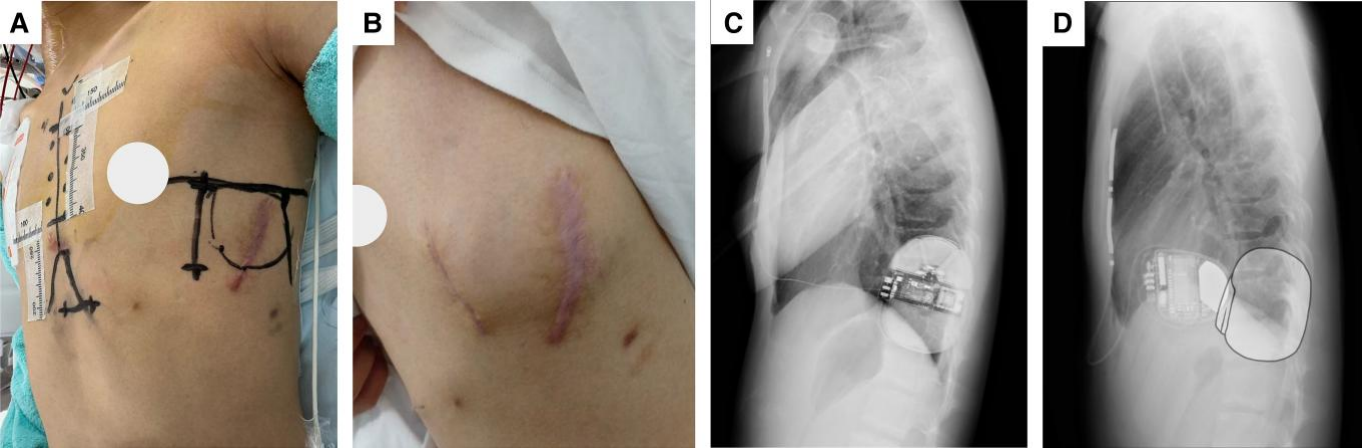
A comparison of the generator positionings between EV-ICD and S-ICD. Pictures of the pre-operative implantation design (*A*) and the post-operative lateral chest incision site after 1 month (*B*) illustrate the relationship between the previous S-ICD generator pocket site and the EV-ICD generator implanted at the recommended position determined by the left-most and posterior wall silhouettes of the left ventricle in the anterior–posterior and left lateral fluoroscopic views, respectively. A comparison of left lateral chest radiographs prior to S-ICD extraction (*C*) and after EV-ICD implantation (*D*) suggests partial overlap between the previous S-ICD generator pocket site and the newly implanted EV-ICD generator position, as well as portions of the lead pathway. EV-ICD, extravascular implantable cardioverter-defibrillator; S-ICD, subcutaneous implantable cardioverter-defibrillator.

Given his young age and small body size owing to a history of a growth disorder (height, 165 cm; body weight, 50 kg; body mass index, 18.4 kg/m^2^), which was treated with growth hormones, EV-ICD implantation was considered optimal. This technology enables the creation of a new incision at a sufficient distance from the previously infected incision site (*Figure 4A*), thereby preventing the need for subcutaneous lead implantation above the sternum, where the skin is extremely thin. The substernal lead was successfully implanted with an acceptable R-wave amplitude of 3.8 mV on the ring 1–ring 2 vector without P-wave oversensing. The generator was placed at the recommended position.^12^ As a result, the newly created generator partially overlapped the previous S-ICD generator pocket (*Figure 4B–D*). The sensing parameters were satisfactory, and defibrillation testing was successful with a 15 J shock. Eight months after the implantation, an appropriate shock was delivered, and ventricular fibrillation was successfully terminated. No reinfection was observed during the 13-month follow-up period (*Figures 3B* and *4B*). On the ring 1–ring 2 vector, an R-wave amplitude and an impedance remained within acceptable values of 7.7 mV and 342 Ω, respectively.

## Discussion

### Main findings

This case series highlights the utility and feasibility of EV-ICD implantation following infection of a TV-ICD or S-ICD in two patients. The main findings from each case are as follows: (i) EV-ICD generator implantation between the serratus anterior and latissimus dorsi muscles may be beneficial and feasible for patients with a smaller body size and extremely thin skin; and (ii) in the case of an EV-ICD implantation following an S-ICD extraction, the recommended EV-ICD generator implantation site can partially overlap the previously implanted and subsequently extracted S-ICD generator site, despite relatively anterior positioning.

### EV-ICD and infections

Despite the similar total infection rates between TV- and S-ICDs, intravascular lead implantation is associated with a higher risk of endocarditis than subcutaneous lead implantation.^1–5,13^ An EV-ICD system, with a smaller generator size compared with the S-ICD system and substernal lead implantation technology,^7^ has recently gained attention as a novel alternative for patients at high risk of infection. The Pivotal study has reported that infections related to EV-ICD are predominantly localized to the pocket or incision site, with no cases of mediastinitis, suggesting a relatively lower risk of severe bloodstream infection.^14^ These characteristics make EV-ICD a replacement device following a TV- or S-ICD infection. To our knowledge, this is the first report of an EV-ICD implantation technique for the optimal conversion of TV- and S-ICD systems resulting from infections.

### Intermuscular EV-ICD implantation following TV-ICD infection

Concerning Patient 1, with TV-ICD generator exposure because of extremely thin skin, intermuscular EV-ICD implantation was performed to reduce the risk of recurrence of exposure and infection, resulting in posterior generator placement relative to the recommended position. This implantation design and technique has been utilized for S-ICD recipients^11^ and for paediatric recipients of the EV-ICD.^15,16^

One simulation study suggested that better EV-ICD defibrillation performance may be achieved at the location determined by the left-most and posterior wall silhouettes of the left ventricle in the anterior–posterior and left lateral views, respectively.^12^ For Patient 1, despite a relatively posterior generator positioning compared with the previous study recommendation, the induced ventricular fibrillation was successfully defibrillated with a 25 J safety margin. While further investigations are needed, this implantation position has the potential to achieve better defibrillation performance, even in EV-ICD, as the intermuscular space between the serratus anterior and latissimus dorsi muscles involves a small amount of fat.^17,18^ The intermuscular EV-ICD implantation technique may be feasible and useful in selected patients.

### EV-ICD implantation following S-ICD infections

For Patient 2, the difference in midline incision positioning between the S-ICD and EV-ICD was clinically relevant. While the midline incision for S-ICD lead implantation is typically placed cranial to the xiphoid process, EV-ICD lead implantation requires a more caudal incision for a safe substernal entry. This difference allowed the avoidance of a direct overlap with the previously infected midline incision. However, a comparison of pre- and post-procedural chest radiographs in this case showed that the lateral generator pocket and lead pathway extending from the midline incision partially overlapped within the lateral thoracic region (*Figures 3A–B* and *4C–D*). When converting from an S-ICD to an EV-ICD owing to infection, careful consideration of the implant design and the timing of reimplantation based on the infected sites is required. Particularly in cases of lateral S-ICD generator pocket erosion, the previously reported shift-and-cover technique, which shifts the pocket from intermuscular to submuscular, can be an alternative strategy.^19^

## Limitations

This case series has some limitations. It comprised only two patients, limiting the generalizability of the findings. The follow-up duration was relatively short, and long-term outcomes regarding reinfection and device performance require further observation. The intermuscular implantation approach used for Patient 1 resulted in generator placement outside the positions suggested in simulation studies, and the balance between infection prevention and defibrillation efficiency requires further investigation. The decision to select an EV-ICD in both cases was patient specific, and EV-ICD implantation could not be uniformly recommended for all patients after device infection. This case series provides practical insights into individualized reimplantation strategies for challenging clinical scenarios.

## Conclusions

Implantation of an EV-ICD following extraction of an infected ICD device is feasible. Individualized reimplantation strategies are required based on patient characteristics and the cause of previous device infection.

## Data Availability

The data underlying this article are not available due to ethical restrictions.

## Lead author biography

Satoshi Ono, MD, graduated from Yokohama City University School of Medicine, Japan, in 2017. After joining the Department of Cardiology at Yokohama City University, he completed his residency training in internal medicine and cardiology at university-affiliated hospitals in Kanagawa Prefecture. He is currently undergoing subspecialty training in arrhythmia management and cardiac electrophysiology as a clinical fellow in the Division of Arrhythmia and Electrophysiology at the National Cerebral and Cardiovascular Center.

## Notes

**Consent:** The authors confirm that written consent for the submission and publication of this case series, including images and associated text, has been obtained from the patients in line with the COPE guidelines.
